# Perspective-Taking in Sentence Comprehension: Time and Empathy

**DOI:** 10.3389/fpsyg.2018.01574

**Published:** 2018-08-29

**Authors:** Shingo Tokimoto, Naoko Tokimoto

**Affiliations:** ^1^Department of English Language Studies, Mejiro University, Tokyo, Japan; ^2^Department of Policy Management, Shobi University, Kawagoe, Japan

**Keywords:** perspective-taking, empathy, dipole fitting, EEG, event-related potential, time-frequency analysis, IC clustering

## Abstract

This study examines the neural substrate of perspective-taking by analyzing the electroencephalographic (EEG) activity elicited by the auditory comprehension of sentences for which the comprehender had to adopt the perspective of the person described in them. Recent studies suggest that the ability of perspective-taking can be an integrative function of temporal and spatial information processing. We thus examined the independence and possible interaction of human perspective shifts and temporal perspective-taking by utilizing Japanese subsidiary verbs for giving, namely *-ageru* and *-kureru*. We manipulated human perspective shifts and temporal perspective-taking independently in experimental sentences by syntactically changing the subject and the object between the speaker and a third person, while we manipulated the tense to be past or non-past tense via sentence-final particles *ru/ta* (non-past/past). The EEG analyses via electrodes indicated the suppression of the β band for human perspective shifts in sentences in non-past tense and the absence of such suppression in sentences in past tense. The analyses for the clusters of independent components indicated β suppression for past tense against non-past tense in sentences without a human perspective shift. This response pattern suggests a close relationship between human perspective shifting and temporal perspective-taking. The β suppression for the human perspective shift in our experiment can be understood as a replication of the previous EEG findings observed for perspective-taking in the presentation of visual images. The preceding findings and our result suggest that the ability or the function of perspective-taking is not specific to the modality. Furthermore, the generator of the β suppression for past tense against non-past tense without human perspective shifting was localized in the precuneus, which is consistent with recent findings indicating that the precuneus is deeply involved in time perception.

## 1. Introduction

Psychologists and neuroscientists are devoting considerable attention to social cognition (Frith and Frith, 2007). Perspective-taking is the act of perceiving a situation or understanding a concept from an alternative point of view, which is an essential component of social cognition, namely, empathy. Many researchers have examined the neural substrate of perspective-taking. Mano et al. (2009) presented short narratives of two sentences to participants in an MRI scanner and recorded brain activity when they were reading them. Mano et al. (2009) manipulated the spatial perspective of the protagonist in their narratives. That is, in their spatially coupled (SC) condition, the first sentence (S1) and the second (S2) described events that occurred at the same place; therefore, the protagonist would be aware of both events, as exemplified in (1).

(1) Spatially Coupled (SC) Condition
  S1: Kana is playing with her much-loved stuffed toy.
  S2: Kana's stuffed toy was pecked and ripped by a bird in her room.

In their spatially decoupled (SD) condition, on the other hand, the events described in S1 and S2 were spatially separated; therefore, the protagonist was not aware of the event described in S2, as in (2).

(2) Spatially Decoupled (SD) Condition
  S1: Kana is watching her favorite comedy at the movie theater.
  S2: Kana's stuffed toy was pecked and ripped by a bird in her room.

Participants were required to evaluate the emotional state of the protagonist portrayed in S2. The protagonists in S1 and S2 were the same in the SC and SD conditions, whereas they were different in the control condition. The assumption was that a “here and now” perspective is adopted as the default view to understand the narratives, whereby information spatially close to the protagonist would be more readily accessible than spatially separate information (Bower and Morrow, 1990). However, if the protagonist was in a different location from the reader's perspective (“there and now”), the reader had to adopt an allocentric perspective to achieve emotional comprehension. Their experimental results indicated that the areas involved in perspective-taking represented by the SD-SC contrast were the precuneus extending to posterior cingulate cortex and the right temporo-parietal junction (TPJ).

Komeda et al. (2016) noted that the narratives in Mano et al. (2009) potentially involved temporal perspective-taking in addition to spatial perspective-taking. Komeda et al. (2016) thus performed an fMRI experiment to examine the relationship between temporal and spatial processing in perspective-taking by manipulating time (viz., the presence or absence of time passage) and location (viz., same or different) independently in the experimental narratives. The experimental stories were presented to 21 typically developing (TD) adults and 20 adults with autism spectrum disorder (ASD) in an MRI scanner. In the different-location condition, the right TPJ was more activated in the ASD group than in the TD group. The right TPJ has been found to be related to spatial perspective-taking (Ferstl and von Cramon, 2007). In the time-passage condition, on the other hand, the anterior cingulate cortex (ACC) was more activated in the TD group than in the ASD group. The ACC is activated in time perception, especially when participants compare long- and short-interval estimation (Pouthas et al., 2005). Komeda et al. (2016) suggested that perspective-taking was an integrated function of temporal and spatial information processing.

The question here is the independence of the shift of human perspective, and we should examine the presence of neural activity particular to it. The shift of human perspective should thus be manipulated independently from temporal and spatial information. Two-sentence narratives are often discussed in experimental studies on perspective-taking. A simple sentence includes at least one proposition and describes one event. It is therefore difficult to manipulate the shift of human perspective alone in two-sentence narratives while keeping the story and description plausible and consistent. In the current study, therefore, we independently manipulated the shift of human perspective and temporal perspective-taking utilizing the Japanese verbs for giving *ageru* and *kureru*.

The literal meaning of *ageru* and *kureru* is to give, and they can function as subsidiary benefactive verbs to constitute various compound verbs. For example, both sentences in (3) including *-ageta* and *-kureta* imply that the lesson in golf was beneficial to the recipient (*Hanako*) (with *-nom, -dat*, and *-acc* being abbreviations for the nominative, dative, and accusative cases, respectively). Beyond these two common features, the two sentences are different with respect to whose point of view the speaker is representing. That is, in (3-a), *ageta* indicates that the speaker described the event from the point of view of the subject (*Taro*), whereas in (3-b), *kureta* indicates that the speaker did so from the point of view of the *ni*-marked object (*Hanako*).

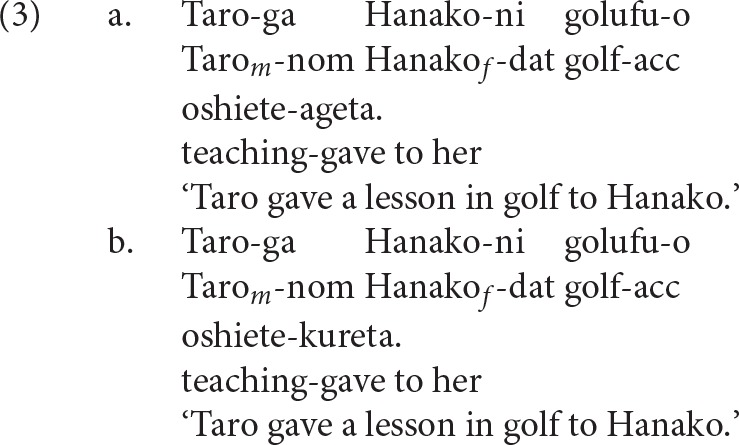



Kuno (1987) metaphorically described the point of view as “camera angle” and defined it as linguistic empathy in (4).

(4) Linguistic empathy
  Linguistic empathy is the speaker's identification, which may vary in degree, with a person/thing that participates in the event or state that s/he describes in a sentence^1^.

Japanese has several other words and constructions representing linguistic empathy, and one of them is the first-person singular pronoun *watashi*. Kuno (1987) characterized the effect of *watashi* on linguistic empathy in (5).

(5) Speech act empathy hierarchy
  The speaker cannot empathize with someone else more than with himself/herself.

The effects of *ageta* and *kureta* on linguistic empathy can interact with that of *watashi*. In (6-a), *watashi* and *-ageta* both indicate that the speaker is describing the event from the view point of the speaker (*watashi*). In (6-b), again, *watashi* and *-kureta* indicate that the speaker is describing the event from the view point of the speaker.

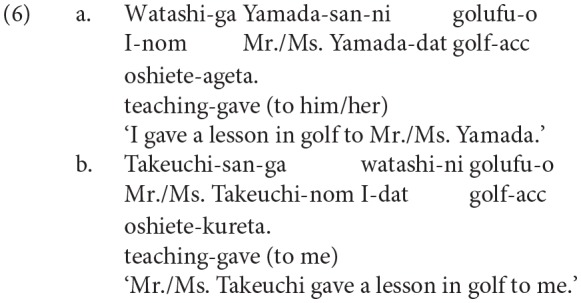



On the other hand, it seems that Japanese native speakers never produce the examples in (7) as natural Japanese sentences, where the subject and the *ni*-marked object are replaced.

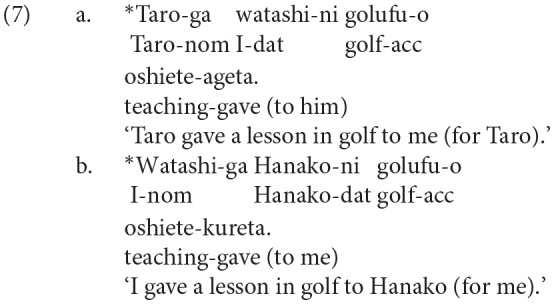



The unnaturalness of (7) can be explained by the conflict of linguistic empathy. That is, in (7-a), the speaker cannot empathize with *Taro* because the sentence includes the word *watashi*, referring to the speaker, whereas *ageta* indicates the speaker's identification with *Taro*. In the same manner, in (7-b), due to the presence of *watashi*, the speaker cannot empathize with *Hanako*; in contrast, *kureta* indicates the speaker's identification with *Hanako*. Kuno (1987) generalized this constraint on linguistic empathy in (8).

(8) Ban on conflicting linguistic empathy foci
  A single sentence cannot contain logical conflicts in empathy relationships.

The constraint in (8) is imposed on the production of a sentence by a speaker. A speaker describes an event from the point of view of her/himself by default, and s/he can make an utterance from the perspective different from hers/his. Here, we can judge that the constraint in (8) is shared with hearers and readers because they can notice the unnaturalness of (7). Linguistic empathy is assumed to be the speaker's identification with the person (or the thing) in a sentence that the speaker produces; therefore, we can manipulate a human perspective-shift in sentence comprehension by changing the linguistic empathy with the assumption that the speaker's linguistic empathy is shared by the comprehenders. For example, in (9), the speaker represents his/her identification with *otooto* (brother) by *ageta* in (9-a) and with *chichi* (father) in (9-b).

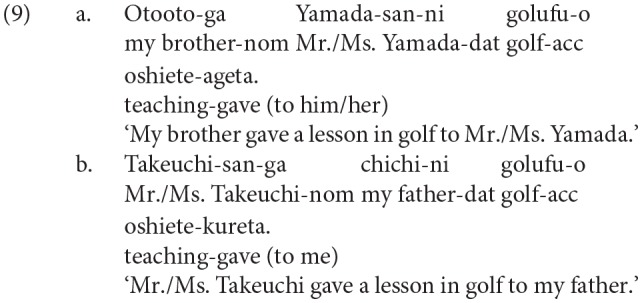



A comprehender will take a perspective-shift from the speaker to *otooto* in (10-a) and *chichi* in (10-b) because the comprehender shares the speaker's linguistic empathy.

As for the temporal expression, Japanese verbs have two forms corresponding to past or non-past tense. A non-past form can represent an event in the present or in the future. We can change the past tense in (6) and (9) to non-past by replacing the sentence's final *-ta* with *-ru* while maintaining their thematic relationship (i.e., who did what to whom), as in (10).

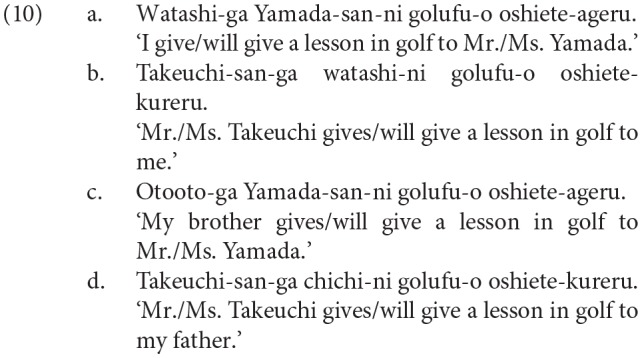



This paper will discuss (6), (9), and (10) as experimental and (11) as control sentences. (11-a) involves an apparent violation of the ban on conflicting linguistic empathy foci because -ageta requires the perspective of the subject [*Kawamura-san* (Mr./Ms. Kawamura)], whereas *watashi* requires that of the speaker. In the same way, *-kureru* in (11-b) requires the perspective of the dative object *Okumura-san* (Mr./Ms. Okumura), and the speaker is assumed to feel empathy for his/her daughter *musune*. It is thus difficult for the comprehender to find a consistent linguistic empathy in (11-b).

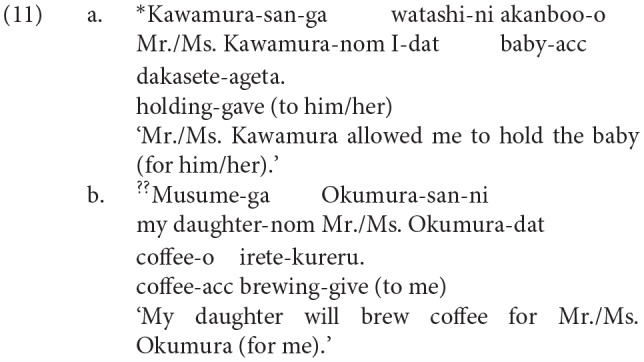



There are electroencephalographic (EEG) and magnetoencephalographic (MEG) experiments on perspective-taking that devote particular attention to time-frequency spectra (Hari et al., 1998; Sakihara et al., 2012; Woodruff et al., 2016). Woodruff et al. (2016) presented participants with a series of photographs of happy, sad, angry and neutral faces. The participants were required to indicate which of the four emotions was being displayed in each photograph in two blocks (other-condition) and to indicate how the emotion of the actor makes the participant feel (self-condition) in a different set of two blocks. Woodruff et al. (2016) observed β suppression at the F3 and C3 electrodes for the other-condition and β enhancement at the F4, Fz, C3, C4, and Cz electrodes for the self-condition. Woodruff et al. (2016) contended that β enhancement and suppression arose separately from distinct neural substrates. Regarding the topography of electrophysiological brain activity, Beck et al. (2017) examined level 1 visual perspective-taking (VPT) by presenting visual scenes that depicted a person and an object in a room using the ‘fast periodic visual stimulation’ technique. Level-1 VPT is one of the earliest manifestations of a child's development in the area of perspective-taking (Sodian et al., 2007), and higher primates, such as chimpanzees, might share this perspective-taking ability with humans (Hare et al., 2000). Beck et al. (2017) created two different sets of stimuli. The objects were always placed in front of the person on either the right or the left wall in the scene for the first set as the consistent perspective stimuli, where the person and the participant saw the object. For the second set of stimuli, on the other hand, the objects were always placed on the wall behind the person in the scene as the inconsistent perspective stimuli. Here, the person and the participant had a different visual experience because only the participant could see the object. Beck et al. (2017) examined the signal-to-noise ratio for the two types of stimuli and observed significant contrasts between them in the right prefrontal and the centro-parietal regions. Beck et al. (2017) suggested that the centro-parietal topography might be generated by the processes in temporo-parietal brain areas involved in the representation of the other person's perspective (Aichhorn et al., 2006; Van Overwalle, 2009; McCleery et al., 2011) and highlighted the role of the right prefrontal cortex in inhibiting our own point of view to allow the selection of the other person's perspective when the two perspectives are in conflict (Vogeley et al., 2001; Samson et al., 2005; McCleery et al., 2011; Shibata and Inui, 2011; Hartwright et al., 2015).

The current study will discuss the neural substrate of perspective-taking by analyzing the EEG activity elicited by Japanese sentence comprehension. We enumerate our research questions in (12).

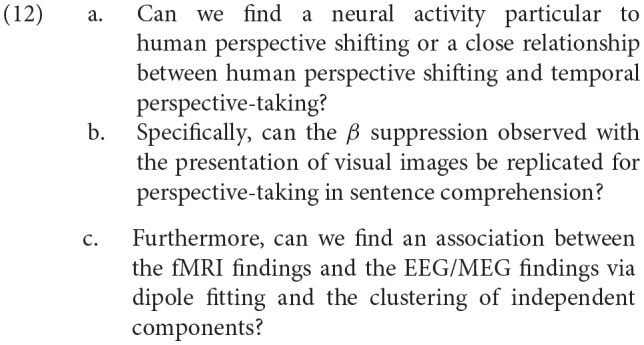



## 2. Materials and methods

### 2.1. Participants

Twenty-one native speakers of Japanese, aged between 20 and 46 years (*M* = 28.67, *SD* = 8.78, 10 males), participated in this study for payment. They had normal or corrected-to-normal vision and had no history of neurological/psychiatric disorders. All the participants were right-handed, as assessed by the handedness questionnaire (Oldfield, 1971). This study was approved by the ethics committee of Mejiro University. Written informed consent was obtained from each participant.

### 2.2. Materials

We created 30 sentences for each of the eight combinations of the three conditions: the presence or the absence of the shift of human perspective, past or non-past form of the verbs, and *-ageru* or *-kureru*. The 240 experimental sentences were counterbalanced to be divided into two stimulus sets, each including 120 sentences. Forty control sentences were included in the main session; therefore, the main session included 160 sentences.

### 2.3. Procedure

The participants were seated in an electrically and acoustically shielded EEG chamber 1 m in front of a 19-inch LCD monitor. A beep sound indicated the beginning of a trial with the visual presentation of a white fixation point at the center of the display. A stimulus sentence was auditorily presented 1 s after the beep. The fixation point turned yellow 1 s after the end of the sentence, and the participants were asked to press the button when they judged the sentence unnatural. The order of the presentation of the stimulus sentences were randomized for each participant. The experiment was controlled using Presentation software (Neurobehavioral Systems). The practice session consisted of 10 trials. The main session consisted of three blocks, and the participants were allowed to rest for 3–5 min between the blocks. The experimental sessions, including instruction and application of the electrodes, lasted ~1.5 h. They completed the Autism-Spectrum Quotient (AQ) in Japanese (Wakabayashi et al., 2006) after their EEG recordings.

### 2.4. EEG recording

EEG signals were recorded using a 32-channel EEG amplifier (Brain Amp DC, Brain Products, Germany) with an active electrode recording system (actiCAP, Brain Products; extended 10–20 montage). The signals were sampled at 2.5 kHz with a band-pass filter of 0.1– 1,000 Hz with the reference electrode positioned at FCz. Vertical and horizontal electrooculograms were simultaneously recorded from electrodes below the right eye (VEOG) and at the outer canthus of the left eye (HEOG). The electrode impedance was maintained lower than 20 kΩ during the sessions. The EEG data were continuously acquired using Brain Vision Recorder software (Brain Products).

### 2.5. EEG data preprocessing

The acquired EEG data were processed offline using EEGLAB (Delorme and Makeig, 2004). The preprocessing proceeded as follows. (1) The EEG data were downsampled to 250 Hz. (2) The data were highpass-filtered at 1 Hz to minimize slow drifts. (3) Their line noise was removed using the CleanLine plugin of EEGLAB. (4) An artifact subspace reconstruction was used to remove high-amplitude artifacts from the EEG data (Mullen et al., 2015). (5) The EEG data were then re-referenced to a common average reference. (6) The data were decomposed using adaptive mixture independent component analysis (AMICA) (Palmer et al., 2007). (7) A best-fitting single equivalent current dipole was calculated for each independent component (IC) to match the scalp projection of each IC source using a standardized three-shell boundary element head model. The electrode locations corresponding to the extended 10–20 system were aligned with a standard brain model (Montreal Neurological Institute). (8) ICs were excluded from further analysis for instances in which the equivalent dipole model was located outside the brain or explained <85% of the variance of the corresponding IC scalp map. Furthermore, ICs were removed that were judged by their frequency spectra to be related to eye blinks, horizontal and vertical eye movements, and electromyography. (9) The data were segmented into time epochs relative to event markers from −1 to 2 s around the markers. (10) The remaining ICs were clustered into five clusters for the cluster analysis, using a k-means algorithm for the dipole locations of the ICs. The number of clusters was determined by two criteria, namely, the number of participants in each cluster and the silhouette values (Rousseeuw, 1987). We first attempted to construct as many clusters as possible to the extent that at least 70% of the participants were included in each cluster, and we found that six clusters always included more than 70% of the participants in each cluster in the 20 clustering repetitions. We then calculated the average silhouette values of all the ICs corresponding to the number of clusters (2–6). A silhouette value indicates the degree of confidence that a certain IC belongs to a certain cluster vs. the other clusters. The silhouette value is 1 when all members of a cluster are identical (best separation), and is −1 in the worst case. The remaining epoched ICs were clustered into five clusters because we obtained the greatest average silhouette value for five clusters in the twenty calculations of the silhouette value.

## 3. Results

### 3.1. Behavioral data: yes-no judgments on control sentences

Multivariate multi-level logistic mixed modeling was employed for the judgments on the control sentences, with the genders and ages of the participants and their subscale scores of AQ (social skill, attention switching, local details, communication, and imagination) as fixed factors and with participant and sentence items as random factors. The modeling was performed using the *glmer* function of the *lme4* package in R (Bates et al., 2015). The effect of social skill was significant (estimate = 0.288, SE = 0.138, *z* = 2.08, *p* = 0.038). Speakers with a greater autistic tendency according to social skill judged more control sentences to be natural.

### 3.2. Electrode analysis

#### 3.2.1. Contrast between experimental and control sentences

**Figure 1:**
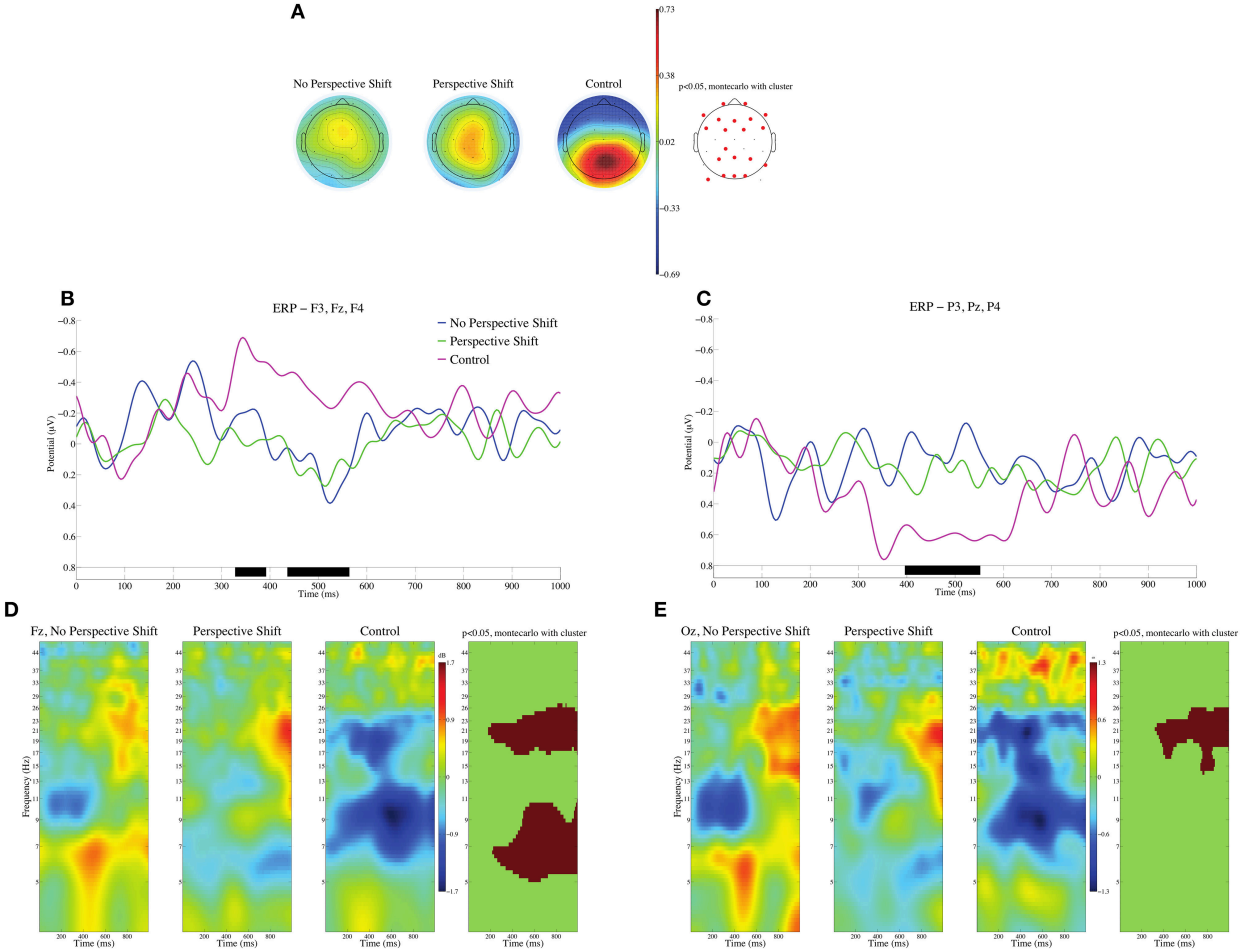
ERPs time-locked to the onsets of subsidiary verbs for giving (*age/kure*) in **(A–C)**. **(A)** Mean topographies of ERPs from 400 to 600 ms for the no perspective shift, perspective shift, and control conditions; electrode sites are depicted in red, and significant differences were found by using the cluster-based permutation test (*p* < 0.05) (Maris and Oostenveld, 2007). **(B)** Mean ERPs of the three conditions at the frontal electrodes (F3, Fz, and F4). **(C)** Mean ERPs of the three conditions at the parietal electrodes (P3, Pz, and P4). Negativity is plotted upward, and the time windows in which significant differences were found by the cluster-based permutation test are indicated in black on the time axis in **(B,C)**. The ERSP at Fz and Oz is depicted in **(D,E)**. The time window and the frequency are represented in red when significant differences were found by using the cluster-based permutation test (*p* < 0.05).

In the processing of the control sentences, their ill-formedness can be assessed at the input of the subsidiary verbs for giving [*age(ru/ta)/kure(ru/ta)*]. Event-related potentials (ERPs) were calculated with the 100 ms after the onset of the subsidiary verbs serving as the baseline. We should note here that the grammatical function of the persons described in the sentences was relevant to perspective-taking. Yokoyama et al. (2009) suggested that it was easier to adopt the perspective of a subject than that of an object. We thus examined the two types of subsidiary verbs as one condition for the grammatical function to avoid confounding the effect of perspective-taking. The mean topographies of ERPs from 400 to 600 ms for the no perspective shift, perspective shift, and control conditions are presented in Figure 1A, and the mean ERPs of the three conditions at the frontal electrodes (F3, Fz, and F4) and the parietal electrodes (P3, Pz, and P4) are presented in Figures 1B,C. Analysis of condition effects was carried out using the STUDY command structure in EEGLAB. To test the significance of condition effects, non-parametric random permutation statistics adopting a 1 × 3 repeated-measures ANOVA design were computed. In the current study, 2,000 random permutations were computed and compared to *F*-values for the mean condition differences. We observed significant deflections for the control sentences, negative in the frontal and positive in the parietal and occipital regions in the time window of 400–600 ms. We found no time window in which the contrast among the three conditions was significant for the HEOG and VEOG electrodes. Event-related spectral perturbation (ERSP) at Fz and Oz are presented in Figures 1D,E. We can observe significant power decreases for the control in the θ, α, and β bands at Fz and in the β band at Oz.

To examine the correlation between the neural activity and AQ scores of the participants, we performed multiple regression analyses following the step-down procedure for the ERP of the control sentences. Because we found a significant negative deflection for the control in the frontal region, we performed a multiple regression analysis for the latency of the minimum of the mean amplitude at the frontal electrodes (F3, Fz, and F4) for the control in the time window of 300–600 ms with the five subscale scores of AQ as independent variables. A marginally significant multiple regression equation was obtained [*F*_(2, 18)_ = 2.74, *p* = 0.091, *R* = 0.43]; the effect of communication was significant [*t* = 2.12, *p* = 0.048, partial regression coefficient (β hereinafter) = 20.69, standardized β = 0.63], and that of attention switching was also significant (*t* = −2.21, *p* = 0.040, β = −21.98, standardized β = −0.65). We performed another multiple regression analysis for the latency of the maximum of the mean amplitude at the parietal electrodes (P3, Pz, and P4) for the control in the time window of 400–600 ms with the five subscale scores of AQ as independent variables, because we found a significant positive deflection in the parietal region. A significant multiple regression equation was obtained [*F*_(2, 18)_ = 3.78, *p* = 0.043, *R* = 0.54]; the effect of attention switching was significant (*t* = 2.52, *p* = 0.022, β = 13.85, standardized β = 0.54), and the effect of imagination was marginally significant (*t* = −2.03, *p* = 0.058, β = −15.17, standardized β = −0.44).

#### 3.2.2. Effects of human perspective shift and temporal perspective-taking

**Figure 2:**
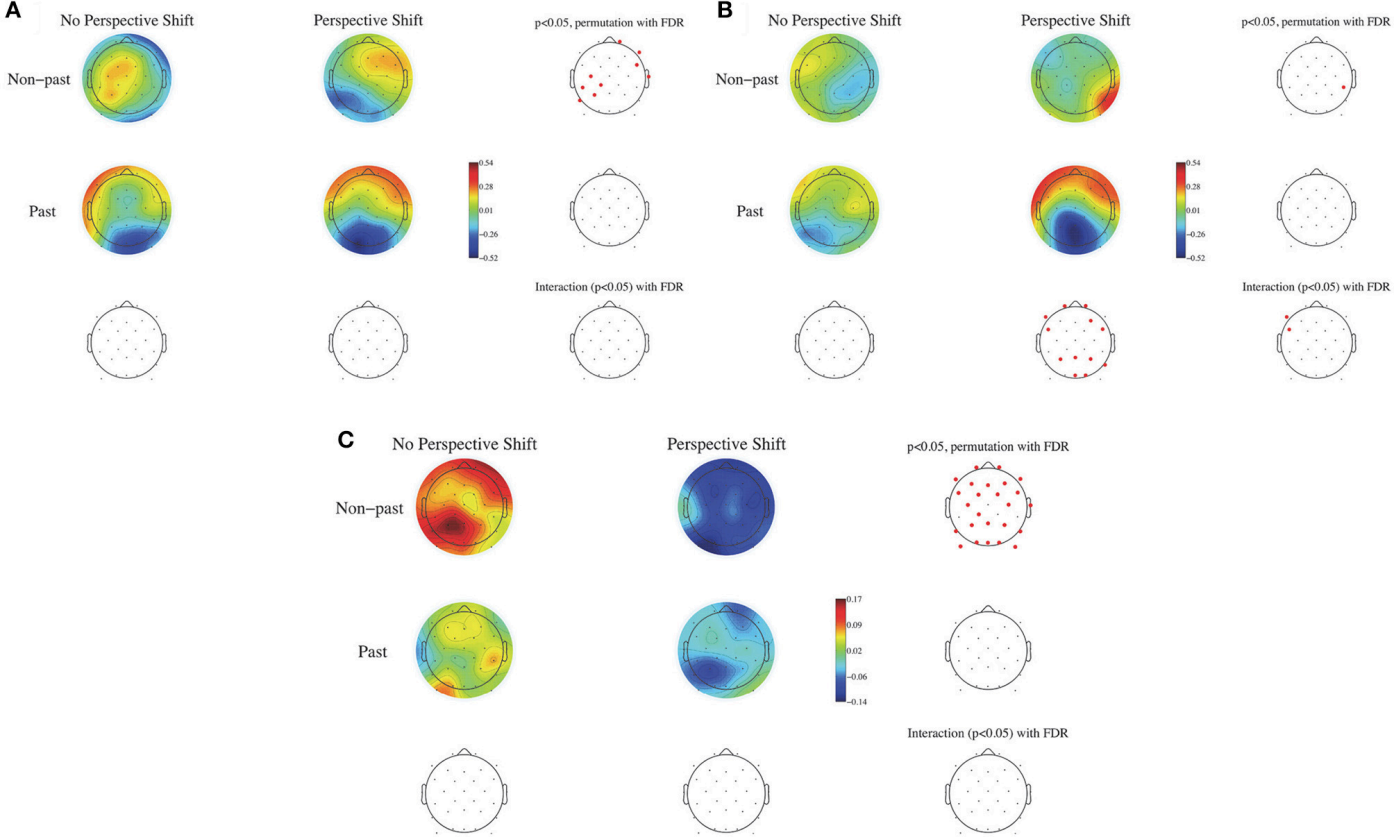
The mean topography of ERPs for the onset of sentence-final particles (*ru/ta*) from 600 to 700 ms in **(A)** and from 800 to 900 ms in **(B)**. The mean power spectral density for the β band (14–28 Hz) in **(C)** for perspective shift and tense. Electrode positions colored in red represent sites showing a significant difference with the use of the FDR correction (*p* < 0.05).

To examine the effects of human perspective shifting and tense and the possible interaction between them, the EEG data were analyzed in a two-by-two factorial design with perspective shift (no perspective shift vs. perspective shift) and tense (non-past vs. past tense) as within-subjects factors. ERPs were calculated time-locked to the sentence-final particles (*ru/ta*), with the baseline being 100 ms after the onset of the particles. Non-parametric random permutation statistics adopting a 2 × 2 repeated-measures ANOVA design were computed using the STUDY command structure for the mean ERPs for every 100-ms time window and for the mean power spectral density in the θ, α, β, and γ bands, with perspective shift and tense as within-subjects factors (with the power spectral density calculated for each participant by subtracting the individual participant mean spectrum). The mean topographies of ERPs from 600 to 700 ms and from 800 to 900 ms are presented in Figures 2A,B, respectively, and the topographies of the power spectral density of the β band (14–28 Hz) are presented for the two conditions in Figure 2C. The multiple comparisons were corrected using the false discovery rate (FDR). A significant effect of perspective shift was observed in the ERP deflections; the effect was positive in the right frontal and negative in the left centro-parietal regions in the time window of 600–700 ms for non-past-tense sentences (Figure 2A). Significant effects of past against non-past tense were observed in the ERP deflections for perspective shift sentences; they were positive in the frontal and negative in the left parietal and occipital regions in the time window of 800–900 ms (Figure 2B). A significant interaction of perspective shift and tense was found in the left frontal region for ERPs in the window of 800–900 ms (Figure 2B). A significant effect of perspective shift was also found in the form of β suppression for non-past-tense sentences (Figure 2C).

We performed multiple regression analyses for the ERP to examine the correlation between the neural activity under the experimental conditions and the AQ scores of the participants. To address the negative deflection for perspective shift in non-past-tense sentences in the left centro-parietal region, we performed a multiple regression analysis following the step-down procedure for the minimum of the mean amplitude of P7, P3, CP5, C3, and CP1 in the time window of 600–700 ms with the five subscale scores of AQ as independent variables. We obtained a significant multiple regression equation [*F*_(3, 17)_ = 6.40, *p* = 0.004, *R* = 0.73]. The effect of communication (*t* = 2.71, *p* = 0.015, β = 0.08, standardized β = 0.72)] and that of attention switching (*t* = −2.69, *p* = 0.016, β = −0.074, standardized β = −0.65) were significant, and the effect of social skill was marginally significant (*t* = 1.90, *p* = 0.075, β = 0.048, standardized β = 0.41). To address the positive deflection for perspective shift in non-past-tense sentences in the right frontal region, we performed a multiple regression analysis for the latency at the maximum amplitude of the mean amplitude of FP2, F8, FC6, and T8 in the time window of 600–700 ms with the five subscale scores as independent variables. A significant regression equation was obtained [*F*_(1, 19)_ = 4.37, *p* = 0.05, *R* = 0.43], and the effect of communication was significant (*t* = −2.09, *p* = 0.05, β = −6.1, standardized β = −2.09). To address the positive deflection in the frontal region for past sentences with perspective shift, we performed a multiple regression analysis for the maximum of the mean amplitude at FP1, F7, FC5, FP2, F4, and FC6 in the time window of 800–900 ms with the five subscale scores of AQ as independent variables. A marginally significant regression equation was obtained [*F*_(1, 19)_ = 3.30, *p* = 0.085, *R* = 0.38], and the effect of social skill was marginally significant (*t* = −1.82, *p* = 0.085, β = −0.07, standardized β = −0.38).

### 3.3. Cluster analysis

**Figure 3:**
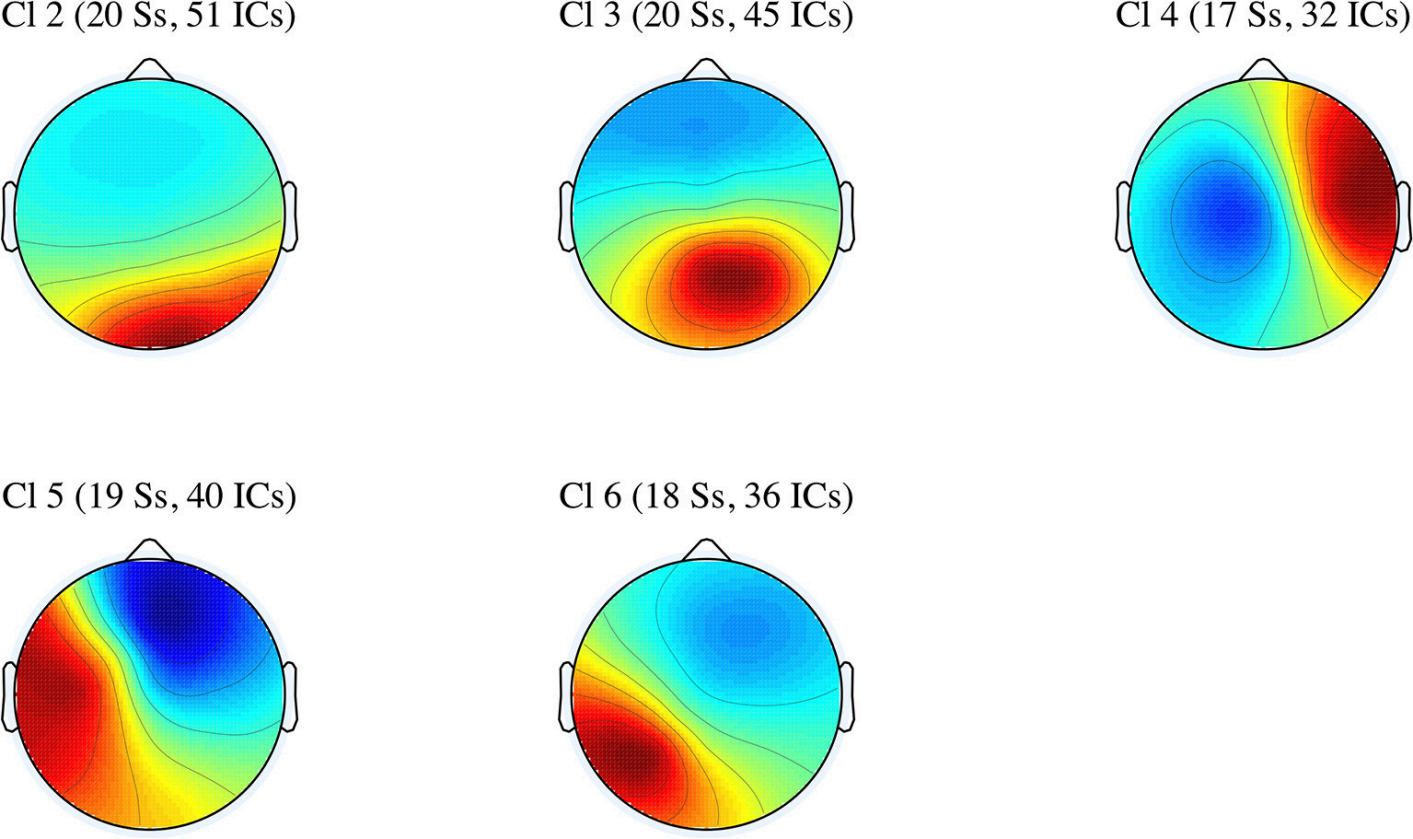
Average scalp maps for the five IC clusters (number of participants and independent components).

In this subsection, we discuss the effects of perspective shift and tense in five IC clusters. Specifically, the differences in the power spectral densities were examined across the five clusters (Cl 2 to Cl 6). The average scalp maps of the five clusters are presented in Figure 3.

The number of ICs included in each cluster, their mean coordinates in Talairach space (Talairach and Tournoux, 1988), their mean residual variances (r.v.), the hemispheres, the structures, and Brodmann areas (BAs) corresponding to the mean coordinates are presented in Table 1.

**Table 1:** The number of ICs included in the five clusters, their mean coordinates in the Talairach space, their mean residual variance (r.v.), the hemisphere, the structure, and the Brodmann areas (BAs) corresponding to the mean coordinates.

	**Number of ICs**	**Mean coordinate**	**Mean r.v. (%)**	**Hemisphere**	**Structure**	**BA**
Cl 2	51	14, −73, −6	3.71	Right	Lingual gyrus	18
Cl 3	45	11, −46, 48	5.89	Right	Precuneus	7
Cl 4	32	36, 5, 35	5.87	Right	Precentral gyrus	9
Cl 5	40	−21, 16, 24	5.13	Left	(Superior frontal gyrus)	(10)
Cl 6	36	−45, −44, 17	4.79	Left	Superior temporal gyrus	13

*The mean coordinate of ICs in Cl 5 is placed in the lateral ventricle, though each IC is localized on the gray matter. The structure and the BA are thus described in parentheses for the IC with the smallest r.v. [0.81%, (x, y, z) = (−5, 59, −1)] in the table*.

#### 3.3.1. Effect of perspective shift in non-past-tense sentences

**Figure 4:**
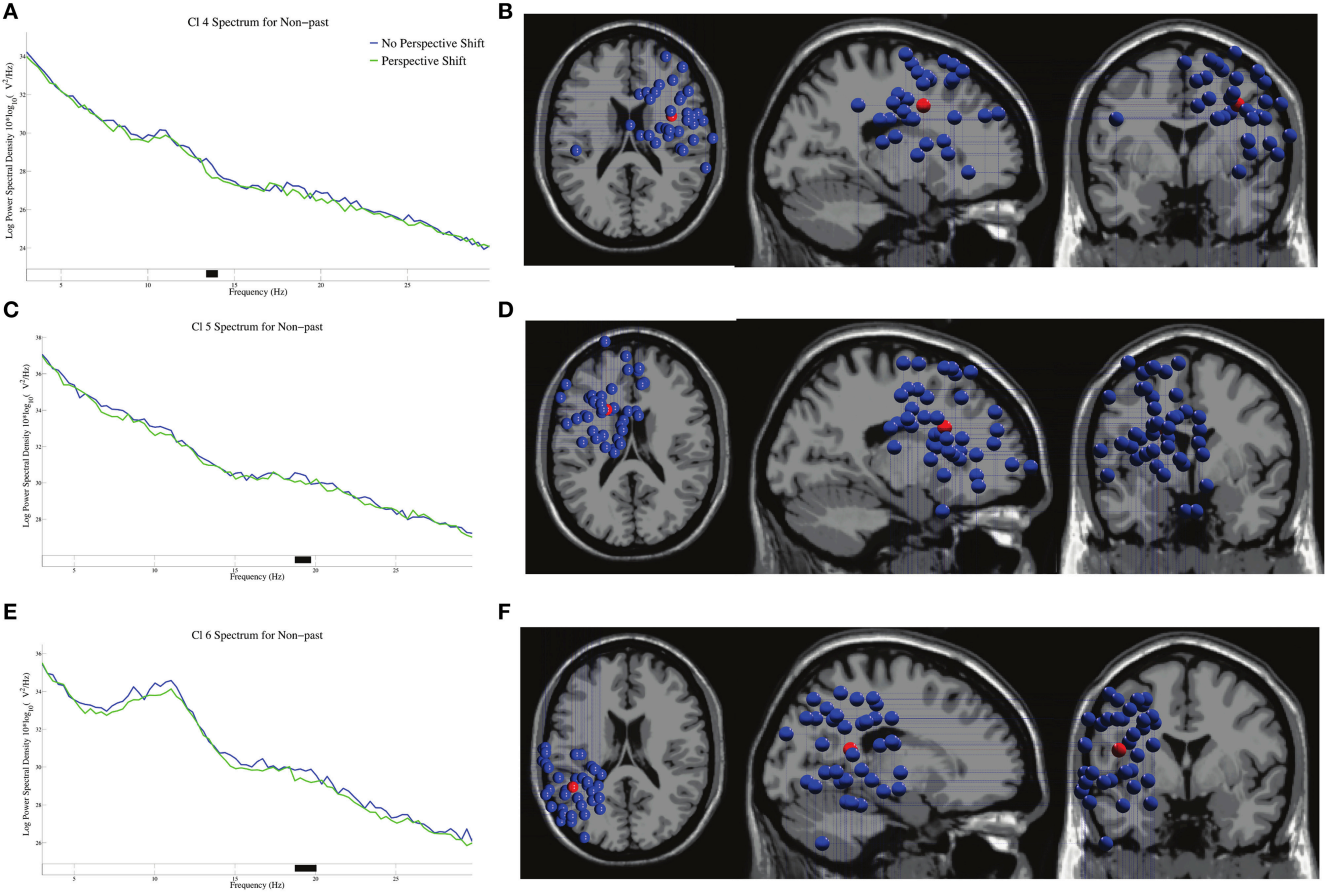
The log-transformed power spectral densities for perspective shift and the dipole locations of Cl 4 in **(A,B)**, Cl 5 in **(C,D)**, and Cl 6 in **(E,F)** in non-past-tense sentences respectively. Black on the frequency axis indicates a significant difference found by the cluster-based permutation test (*p* < 0.05). The red balls in the dipole locations indicate the mean coordinate positions (the same applies hereinafter).

The log-transformed power spectral densities and the dipole locations of Cl 4, Cl 5, and Cl 6 are presented in Figure 4. Significant β suppressions for perspective shift were found at 13–14 Hz in Cl 4, 19 Hz in Cl 5, and 19–20 Hz in Cl 6 in non-past-tense sentences.

#### 3.3.2. Effect of perspective shift in past-tense sentences

**Figure 5:**
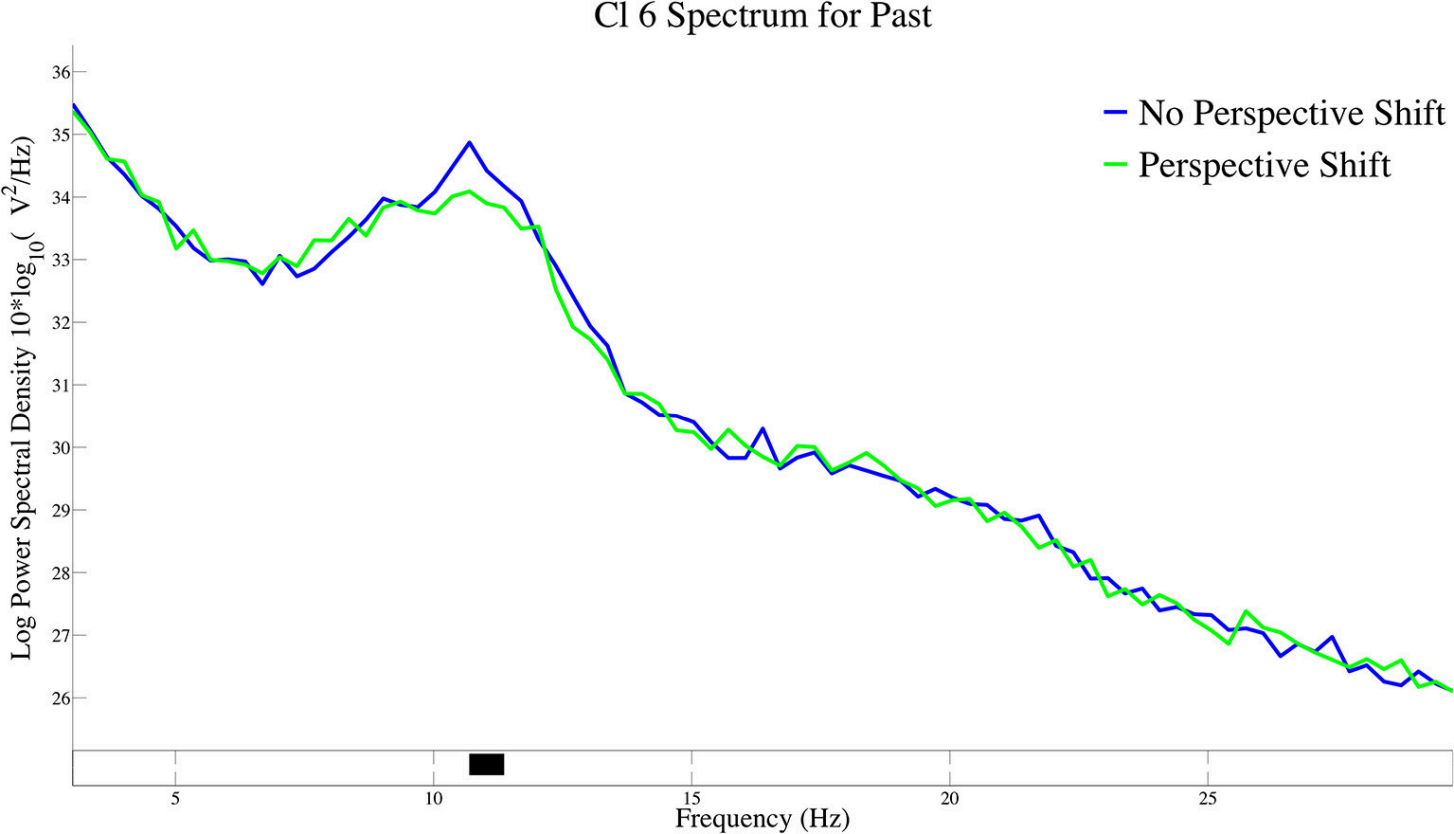
The log-transformed power spectral density for perspective shift in past-tense sentences in Cl 6.

The log-transformed power spectral density for perspective shift in past-tense sentences is presented in Figure 5. A significant suppression for perspective shift was observed at 11 Hz of the power spectral density in Cl 6.

#### 3.3.3. Effect of tense in no-perspective-shift sentences

**Figure 6:**
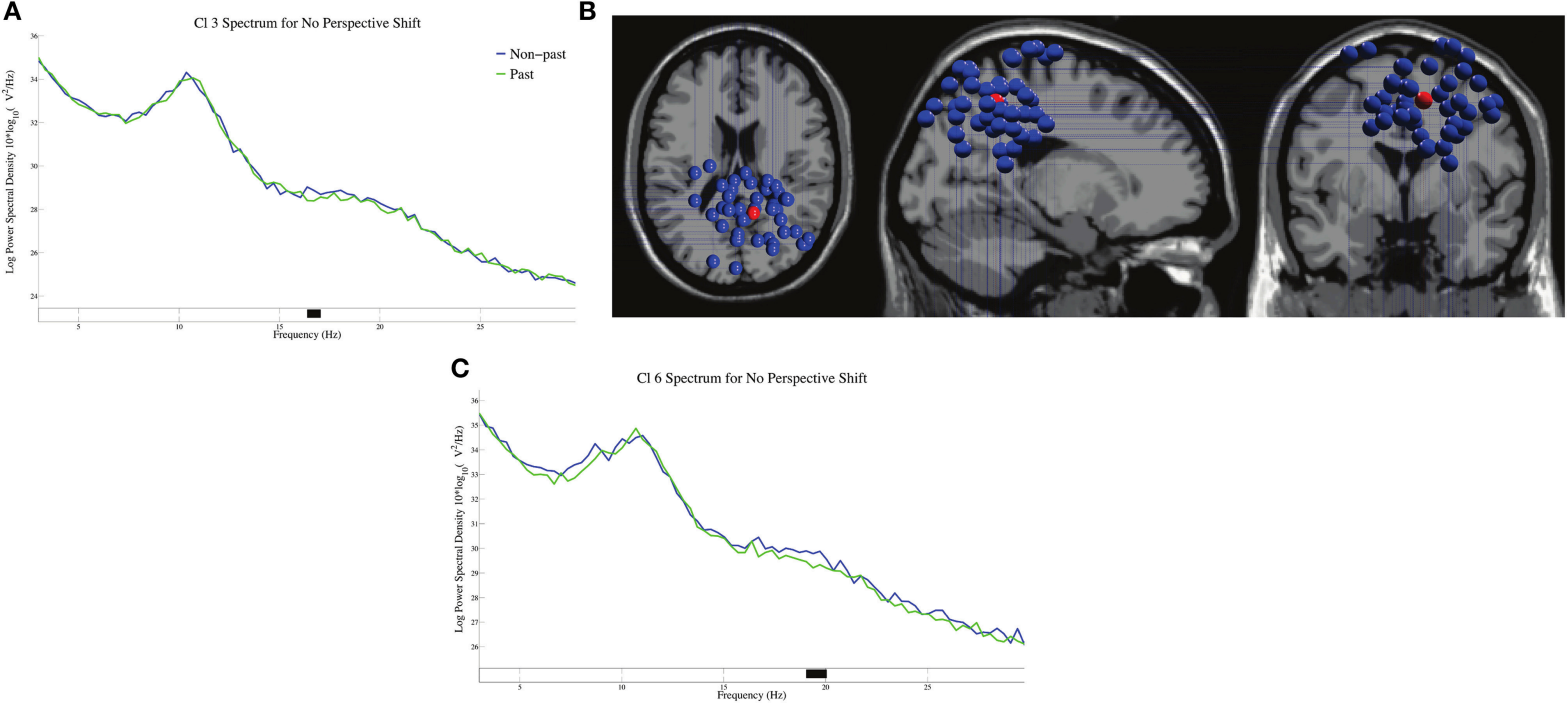
The log-transformed power spectral densities for tense in no-perspective shift sentences for Cl 3 **(A)** and Cl 6 **(C)**, and the dipole locations of Cl 3 **(B)**.

The log-transformed power spectral densities for tense in no-perspective-shift sentences in Cl 3 and Cl 6 are presented in Figures 6A,C. We found significant β suppressions for past against non-past at 17 Hz in Cl 3 and at 19 Hz in Cl 6. The dipole locations in Cl 3 are presented in Figure 6B.

#### 3.3.4. Effect of tense in perspective shift sentences

**Figure 7:**
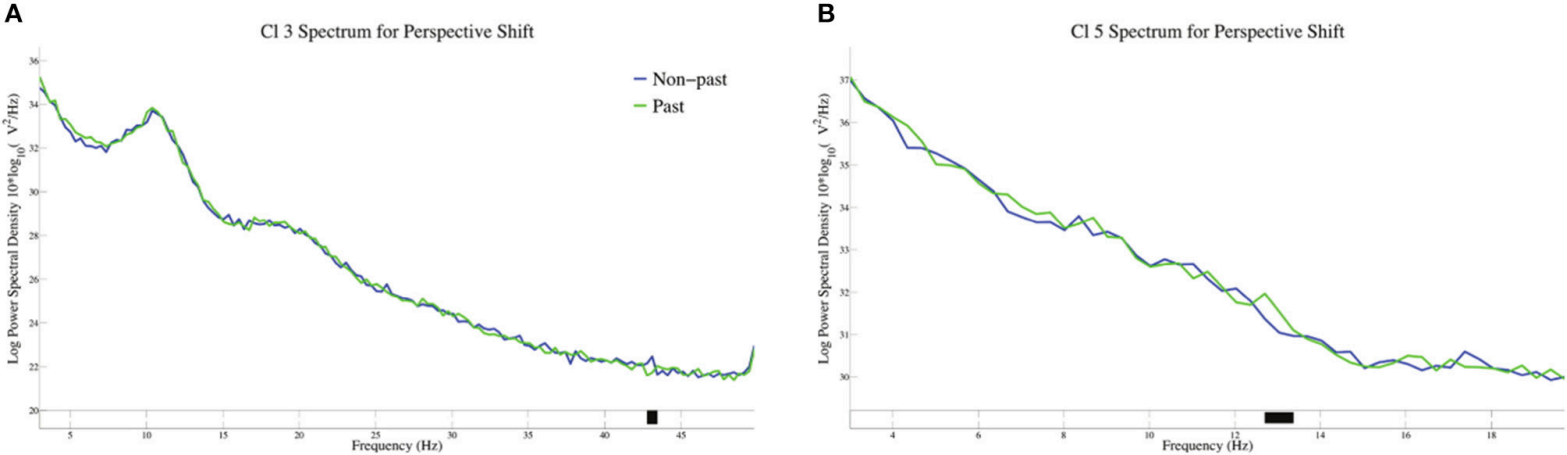
The log-transformed power spectral density for tense in perspective shift sentences in Cl3 **(A)** and Cl5 **(B)**.

The log-transformed power spectral density for tense in perspective shift sentences is presented in Figure 7. A significant suppression for past against non-past-tense sentences was observed at 43 Hz in Cl 3, whereas a significant enhancement for past-tense sentences was found at 13 Hz in Cl 5.

## 4. Discussion

The well-formedness judgments of the control sentences were significantly correlated with a subscale score of AQ. We also obtained significant regression equations for the amplitude and the peak latency of the ERPs for the control and experimental sentences with some of the subscale scores of AQ as independent variables. These results indicate that perspective shifting in sentence comprehension is deeply related to an individual's autistic tendency.

We observed significant contrasts in ERP and ERSP between experimental and control sentences (Figure 1). The topography of ERP for the control sentences is similar to that often observed for linguistically deviant sentences, namely, frontal negativity, and parietal-occipital positivity. However, a power decrease in the β band (Figures 1D,E) is characteristic of our control sentences, whereas the ERPs elicited by a violation of linguistic constraint is often associated with the θ band (Roehm et al., 2004; Hald et al., 2006). The reason for the deviance of our control sentences is the inconsistency of linguistic empathy in a sentence; therefore, the β decrease in ERSP observed for our control sentences can be attributed in part to β suppression for perspective shift, as discussed below.

We observed a broad β suppression for perspective shift in the electrode analysis (Figure 2C). This finding conforms well with the finding for the “other-condition” obtained by Woodruff et al. (2016). To the best of our knowledge, the current study is the first to observe EEG β suppression for perspective-taking in auditory sentence comprehension, whereas previous studies (including Woodruff et al., 2016) examined perspective-taking by using visual images. As for the topography of ERP, significant effects of perspective shift were observed in the right frontal and the (left) centro-parietal regions (Figures 2A,B), which is consistent with Beck et al. (2017). Beck et al. (2017) suggested that the centro-parietal topography could be a manifestation of processes in temporo-parietal brain areas for representing another person's perspective and that the right prefrontal cortex could inhibit our own point of view to allow for the adoption of another person's perspective. Our cluster analyses indicated β suppression in the right precentral gyrus (Figures 4A,B) and the medial parietal region (Figures 6A,B), which is again consistent with the suggestion of Beck et al. (2017). These prior findings and our results suggest that the ability or function of perspective-taking is not specific to the modality.

The most salient EEG pattern in the current study is the significant β suppression for perspective shift in non-past-tense sentences and the absence of such an effect for past-tense sentences (Figure 2C). A suggestion made by Komeda et al. (2016) is instructive here in that perspective-taking is the integrative manifestation of temporal and spatial information processing. The understanding of an utterance referring to a past event could involve temporal perspective-taking, which could be part of human perspective shifting. If the interpretation of the past tense presupposes human perspective-taking, it is not surprising that we found no effect of perspective shift for past-tense sentences. The β suppression of past in Cl 3 and Cl 6 in no-perspective-shift sentences (Figure 6) is suggestive here. The β suppression for perspective shift in non-past-tense sentences and that for past in no-perspective-shift sentences suggest a close relationship between human perspective-taking and processing of past information.

The neural activity for perspective shift in past tense is of theoretical interest here. The EEG patterns for the possible integration of the two closely related processes are different from β suppression, that is, the α suppression in Cl 6 for perspective shifting in past-tense sentences (Figure 5) and the gamma suppression in Cl 3 and the β enhancement in Cl 5 for past in perspective shift sentences (Figure 7). The effect of past tense in perspective shift sentences appeared 200 ms after that of perspective shift in non-past-tense sentences (Figures 2A,B). The later appearance of the former could be a manifestation of integrative and/or counteractive processes for temporal and human perspective-taking.

Recent studies have demonstrated that the bilateral posterior cingulate cortex and precuneus are deeply involved in time perception (Dušek et al., 2012; Berkovich-Ohana, 2013; Carvalho et al., 2016). The β suppression for past tense in no-perspective-shift sentences in Cl 3 (Figure 6) is relevant here because this suppression can be a direct manifestation of temporal perspective-taking for the past. The distribution of the dipoles in Cl 3 is relatively narrow compared with that in the other clusters, and the mean coordinate of the dipoles in Cl 3 is located in the precuneus (Figure 6B). The β suppression for past tense in no-perspective-shift sentences thus supports previous findings in that the precuneus is deeply involved in time perception.

## 5. Limitations

The ban on conflicting linguistic empathy foci in (8) can be assumed to be cross-linguistic, because the speaker's identification with others will be a universal phenomenon. Kuno (1987) discussed the linguistic phenomena in English and Turkish that could be explained by (8). Furthermore, Kuno (1978) discussed the application of his linguistic-empathy construct to Latin, Greek, Icelandic, and Russian. However, we can find no systematic experimental study that examined the cross-linguistic property of linguistic empathy. At present, therefore, we cannot deny the possibility that the neural activity found in the current study is peculiar to Japanese.

We should be careful in interpreting the source localization by dipole fitting because the distribution of dipoles is relatively wide in some of the clusters. Vigneau et al. (2006) performed a meta-analysis of 129 scientific reports that examined the brain areas involved in language processing and demonstrated that many areas overlapped for phonological, syntactic, and semantic processing. Vigneau et al. (2006) argued for an architecture with large-scale networks rather than modular organization of language in the left hemisphere. In the current study, the distribution of dipoles could be wide, because sentence comprehension simultaneously involved many brain regions. Furthermore, we should, theoretically and empirically, consider the best criterion for IC clustering.

We should also be careful in discussing a mental process on the basis of brain activity. This is because even when we find any brain activity in a region for a task, we cannot definitely conclude that the region is responsible for the mental processes of the task. This is a problem of “reverse inference” (Poldrack, 2006). In the current study, we performed dipole fitting to interpret the experimental findings by integrating the fMRI and EEG data. We believe that our analysis was successful to some extent in that the β suppression observed in the EEG recordings, which corresponds to the finding of Woodruff et al. (2016), was localized in the precuneus, where Mano et al. (2009) observed activation during (temporal) perspective-taking. However, we have not clarified the function of the multiple circuits in perspective-taking. Poldrack (2011) proposed some large-scale decoding of fMRI data (multivoxel pattern analyses and model-based approaches) to settle the problem of reverse inference. Studies utilizing the large-scale decoding of EEG data are still few in number (Jalili et al., 2013; Yoshimura et al., 2017). We should examine the effective connectivity among the IC clusters for a better understanding of the neural substrate of perspective-taking.

## 6. Conclusion

The main research topic of the current study is the independence of human perspective shifting from temporal perspective-taking. Our experimental results suggest that the two types of perspective-taking are closely related to one another. The EEG response patterns observed in previous studies of perspective-taking using the presentation of visual images were replicated in this study in β suppression for perspective-taking and in topography. We believe that this study enhances the potential of using sentence comprehension in examining perspective-taking and empathy. Furthermore, dipole fitting and the clustering of ICs are helpful for integrating the fMRI findings and the EEG/MEG findings.

## Author contributions

ST: design of the work, acquisition of data, analysis, interpretation of data, and manuscript preparation. NT: acquisition of data, analysis, and interpretation of data.

### Conflict of interest statement

The authors declare that the research was conducted in the absence of any commercial or financial relationships that could be construed as a potential conflict of interest.

## Footnotes

^1^Here we refer to the definition of “Empathy” in Kuno (1987) as “linguistic empathy” because the empathy there is restricted to language production.
